# Paradox response of cornea to different color intensities of visible light: An experimental study

**DOI:** 10.1371/journal.pone.0196827

**Published:** 2018-05-25

**Authors:** Sherif S. Mahmoud, Ibrahim H. Ibrahim, Abdel Sattar M. Sallam, Wafaa A. Gareeb

**Affiliations:** 1 Biophysics and Laser Science Unit, Research Institute of Ophthalmology, Giza, Egypt, Giza, Egypt; 2 Physics Department, Faculty of Science, Ain Shams University, Cairo, Egypt, Abbasiya Square, Cairo, Egypt; Bascom Palmer Eye Institute, UNITED STATES

## Abstract

The technological development is associated with human daily life and had an impact on its social life. Due to the difficulty of estimating the daily exposure to light; research is needed to determine how much natural and man-made lights could affect the cornea. Visible light radiation could have damaging effect on the human eye; the type and degree of damage are related to the duration and the cumulative exposure as well as to the intensity of the rays. There are noticeable increases in using electronic devices and colored lamps in decoration and toys as well, without any specific regulation. We studied the effect of such human activity on the corneal structure and the vibrational characteristics of corneal tissue by Fourier transform infrared spectroscopy. To achieve these goals, Chinchilla rabbits were exposed to two different lux of blue, green or red color lamps. The results indicate that the corneal tissue responds non-specifically to each lux and accordingly the color. The detected changes are including corneal protein secondary structure as well as lipids, in particular phospholipids. This was concomitant with more ordered membrane bilayer and changes in the corneal membrane phase organization. No lux/color-response relationship was established.

## Introduction

Human is constantly exposed to a portion of the electromagnetic spectrum where the eye and skin are the primarily target organs [1]. Light is essential for maintaining the circadian rhythm [2] and recently light therapy is included for the treatment of seasonal affective disorder [3]. In addition to the medical applications of optical instruments used in ophthalmic examination, light is used for decoration purposes whether indoor or outdoor. Despite of the risk of exposure to visible light, but without it also has its risk. Phototoxicity of different visible light segments is well known [4–5], and it focuses on the retina with minor attention to the cornea. With the technological evolution, which includes devices such as smart phones and tablets, human life has become closely linked with this technology and, since it is difficult to quantify light exposure through everyday life, this study is devoted to clarify the possible interaction of corneal structural constituents with different light colors (wavelengths)/lux by recording and analyzing the vibrational characteristics using Fourier transform infrared spectroscopy (FTIR).

## Materials and methods

Healthy Chinchilla rabbits weighing 2.5–3 kg were purchased from the holding company for biological products and vaccine, Cairo, Egypt. Rabbits were fed a balanced diet and received water ad-libitum. Use of animals in this study was in compliance with the ARVO statement for the Use of Animals in Ophthalmic and Vision Research, the study was also approved by the Research Institute of Ophthalmology ethical committee (RIO—ethical committee). For irradiation purpose, animals were carefully removed from cages in order to avoid excitement. The potassium bromide-FTIR grade (KBr) powder was purchased from sigma-aldrich (Sigma-Aldrich Co. LLC).

### Visible light exposure

Incandescent lamps (220 V and 60 W) commercially available in the markets were used in this study. Three light lamps (sources) that emit visible light in the blue, green and red regions were calibrated in the National Institute for Standards (Giza, Egypt) in order to determine their illuminance at 10 cm (High lux, H) and 1 m (Low lux, L). The blue lamp (λ_max_ = 415 nm) low lux was 80 (blue-L) and the high lux was 3560 (blue-H). For the green source (λ_max_ = 510 nm) it was 183 (green-L) and 4780 (green-H) lux respectively. The red source (λ_max_ = 610 nm) lux was 162 (red-L) at 1m and 5050 (red-H) at 10 cm. The uncertainty in the measured illumination is ± 3%.

For irradiation purpose, animals were carefully placed in animal's cages so that their heads are exposed. Before irradiation, the eyes were anesthetized by few drops of benox eye drops while the whole rabbits were anesthetized by injection with 0.1 ml/kg separine as muscle relaxant firstly and after 15 min, 0.5 ml/kg ketamine hydrochloride was administered intramuscular. The animal's eye was kept opened during the irradiation using speculum. The light source was adjusted to irradiate the eye by using an obstacle with a hole that was adjusted to fit the diameter of the eye. The thermal effect of light on the exposed eyes was avoided by using infrared filters and water filter, as well as the ambient temperature was set to 20 ^o^C.

Rabbits (n = 60) were categorized into six light-irradiated groups each composed of 10 rabbits; one eye was exposed to either low or high lux of the blue, green or red sources while, the contra-lateral eye was used as control. The exposure time for all lux was 60 min/session and repeated three times daily and lasts for one week.

### Fourier transform infrared spectroscopy

After exposure, eyes were enucleated from the globe and corneas were removed by cutting through the ora serrata. Each cornea was crushed into powder in liquid nitrogen and mortar. The resulted corneal powder was then mixed with KBr powder (10 mg:90 mg) and pressed with the instrument-provided pressing kit to prepare the KBr disks that will be used for the FTIR analysis.

Infrared spectra were recorded using Nicolet iS5 FTIR Spectrometer (Thermo Scientific, USA) equipped with TGS detector. The spectra were taken at 4.0 cm^-1^ resolution at physiological temperature. To avoid the influence of atmospheric CO_2_ and water vapor (if any), the instrument was operated under continuous flow of N_2_ gas. Generally, 100 scans were accumulated to enhance the signal to noise ratio. These interferograms were averaged to produce a representative group spectrum that was analyzed using OriginPro 7.5 software (Origin Lab Corporation, Northampton, MA, USA).

### Statistical analysis

Data were represented by the mean ± SD. In this study the analysis of variance (one way-ANOVA) was used to determine the statistical significance between multiple groups at a significance level of p<0.05.

## Results

Infrared spectral analysis will be performed in the following ranges; 4000–3000 cm^-1^ (amide A and B as well as OH stretching region), 3000–2800 cm^-1^ (CH stretching region), 1700–1600 cm^-1^ (amide I region) and 1500–1200 cm^-1^ (fingerprint region).

### NH-OH stretching region

**Fig 1:**
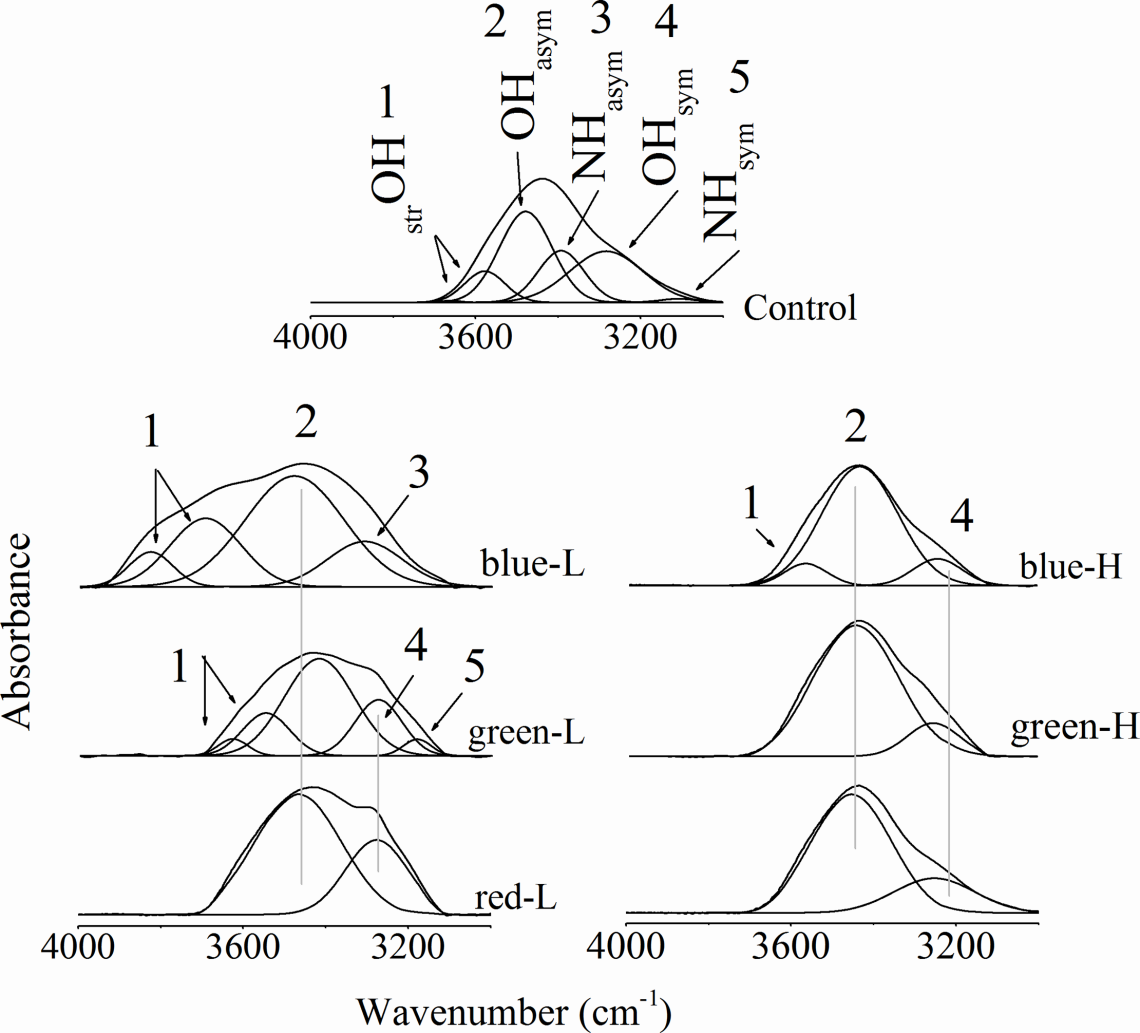
The main NH-OH band and its underlying peaks due to exposure to different color intensities.

The NH-OH region of the corneas that includes Amide A (_asym_NH) and Amide B (_sym_NH) bands is shown in Fig 1. Regarding the main band, the vibrational frequency of control group was found to be 3434 ±1 cm^-1^. After the exposure to light sources, two observations can be noticed: the vibrational frequency was increased due to the exposure to low lux (average, 3446 ± 5 cm^-1^) and remains unchanged after the exposure to high lux (average, 3434 ± 2 cm^-1^) as compared to the control one. The contour of the main band was resolved to its underlying peaks using the curve enhancement-Fourier deconvolution procedure. As shown in Fig 1 and given in Table 1, the number of estimated peaks was decreased after exposure to both low and high lux. The pattern associated with the high lux of the green light (green-H) has the same characteristics as that associated with red-L and red-H; and were characterized by restricted NH stretching vibrational modes as well as the _str_OH one. Table 1 also indicates that Amide A and Amide B bands were dramatically affected by the exposure to different wavelength/lux; Amide A was restricted in all lux but blue-L where its vibrational frequency was significantly decreased and its bandwidth was increased. On the other hand, Amide B band was restricted in all lux but green-L. The band characteristics of the green-L show significant increase in both frequency and bandwidth.

**Table 1:** Underlying peaks detected in the NH-OH stretching region showing their vibrational frequency (cm^-1^) and bandwidth (cm^-1^).

	(1) _Str_OH			(2) OH_asym_	(3) Amide A	(4) OH_sym_	(5) Amide B
Control		3662 ± 3	3577 ± 3	3478 ± 2	3362 ± 2	3283 ± 1	3108 ± 2.4
		10 ± 3	31 ± 6	116 ± 5	58 ± 5	92 ± 4	3.2 ± 1
Blue-L	3828 ± 2	^†^3695 ± 3		3479 ± 2	^†^3309 ± 2		
	105 ± 3	^†^172 ± 10		^†^239 ± 7	^†^182 ± 9		
Blue-H			^†^3567 ± 1	^†^3435 ± 1		^†^3244 ±1	
			^†^104 ± 2	^†^185 ± 5		^†^118 ± 7	
Green-L		^†^3629 ± 3	^†^3548 ± 3	^†^3419 ± 2		^†^3276± 1	^†^3182 ± 1
		^†^71 ± 5	^†^113 ± 3	^†^168 ± 5		^†^123 ± 9	^†^66 ± 2
Green-H				^†^3445 ± 1		3255 ± 2	
				^†^210 ± 8		^†^127 ± 2	
Red-L				^†^3467 ± 2		^†^3276 ±2	
				^†^213 ± 2		^†^150 ± 2	
Red-H				^†^3453 ± 1		^†^3252 ± 3	
				^†^197 ± 6		^†^194 ±4	

^†^Statistically significant.

In each cell, the first line represents the frequency and the second line reflects the bandwidth.

The OH stretching modes (_asym_OH and _sym_OH) have the same characteristics after the exposure to all lux but blue-L. Its vibrational frequency was decreased concomitant with increased bandwidth as compared to the normal. The other OH vibrational mode (_str_OH) was restricted as a result of exposure to green-L, red-L and red-H. The _str_OH vibrational frequency and its corresponding bandwidth were changed due to exposure to blue-L, blue-H and green-L.

### CH stretching region

**Fig 2:**
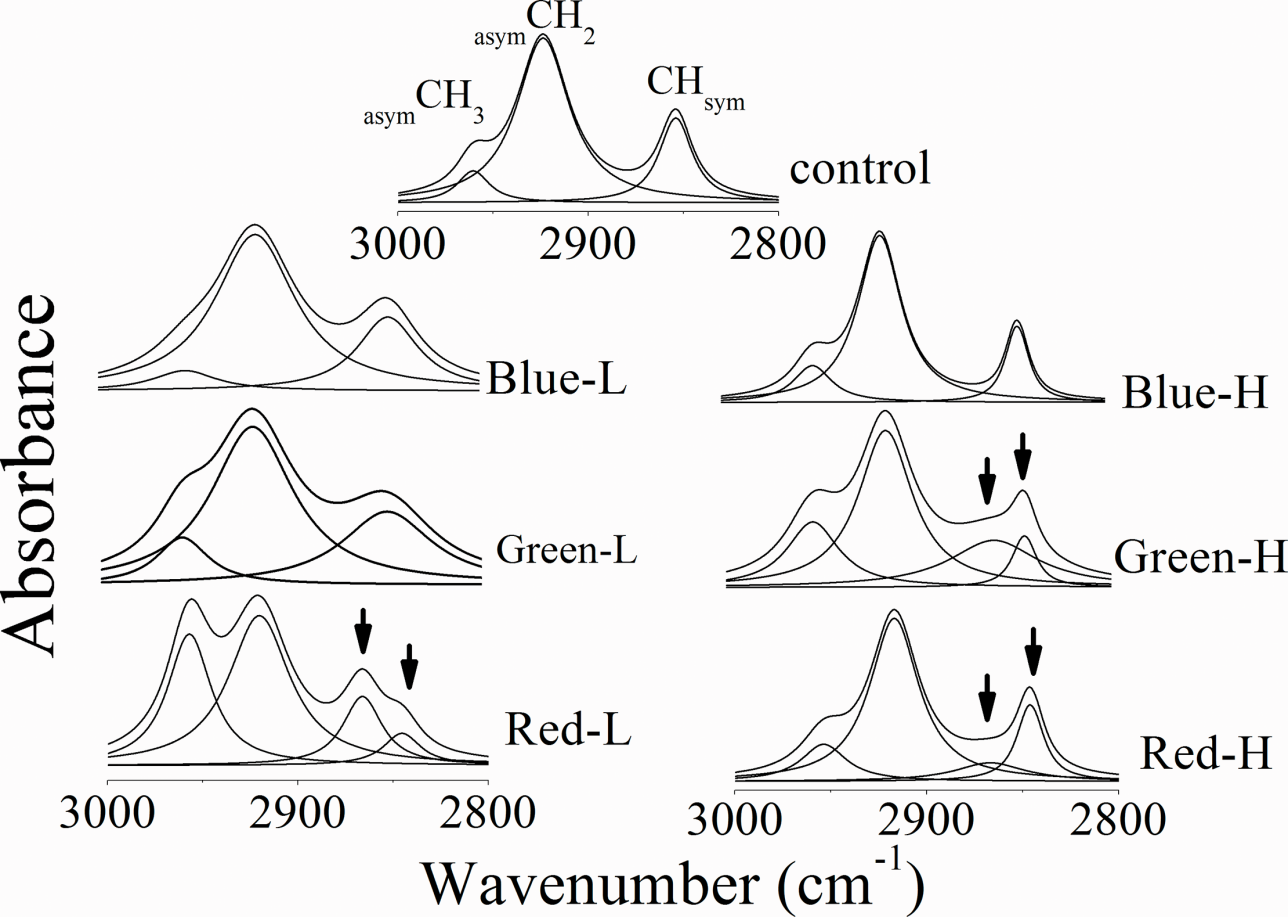
Corneas CH stretching region. Arrows are pointing to the splitting of CH_sym_ mode into _sym_CH_3_ and _sym_CH_2_.

Fig 2 and Table 2 summarize the vibrational characteristics of the CH stretching region in the wavenumber range 3000–2800 cm^-1^. The normal profile indicates the presence of three bands that discernible at 2960 cm^-1^ (_asym_CH_3_), 2923 cm^-1^ (_asym_CH_2_) and 2850 cm^-1^ (_sym_CH). For _asym_CH_3_ mode of vibration, its vibrational frequency was decreased for high lux exposure concomitant with increased vibrational motion for both low and high lux. In the same context, the vibrational frequency of _asym_CH_2_ band was decreased relative to the normal one after the exposure to different light lux. The other observation concerning this band is the increased bandwidth that was associated with low lux only.

**Table 2:** Analysis of the CH region showing the underlying peaks vibrational frequency (cm^-1^) and the corresponding bandwidth (cm^-1^).

	Control	Blue		Green		Red	
		Low	High	Low	High	Low	High
_asym_CH_3_	2960±3	2955±2	^†^2952±2	2957±2	^†^2955±1	2957±2	^†^2953±1
	18±2	^†^40±3	^†^25±2	^†^32±8	^†^32±4	^†^29±3	^†^28±2
_asym_CH_2_	2925±1	^†^2918±2	^†^2919±2	^†^2920±1	^†^2918±2	^†^2920±2	^†^2918±2
	30±4	^†^51±4	28±4	^†^52±5	34±3	^†^41±5	30±3
_sym_CH	2851±2	2849±3	2848±3	2850±2			
	20±5	^†^38±5	^†^13±4	^†^57±2			
_sym_CH_3_					2861±2	2866±2	2867±1
					56±8	24±9	44±6
_sym_CH_2_					2845±1	2845±1	2846±1
					14±3	23±2	15±6

^†^Statistically significant.

In each cell, the first line represents the frequency and the second line reflects the bandwidth.

The detected _sym_CH vibrational modes have no changes in their frequency while their bandwidth was changed. The curve enhancement procedure used in this study, resolved the stretching band that noticed in the normal pattern nearly at 2851 cm^-1^into two underlying component bands due to exposure to red-L, green-H and red-H. These two bands are centered at 2860 cm^-1^ and 2845 cm^-1^ and correspond to _sym_CH_3_ and _sym_CH_2_ respectively.

### Amide I region

**Fig 3:**
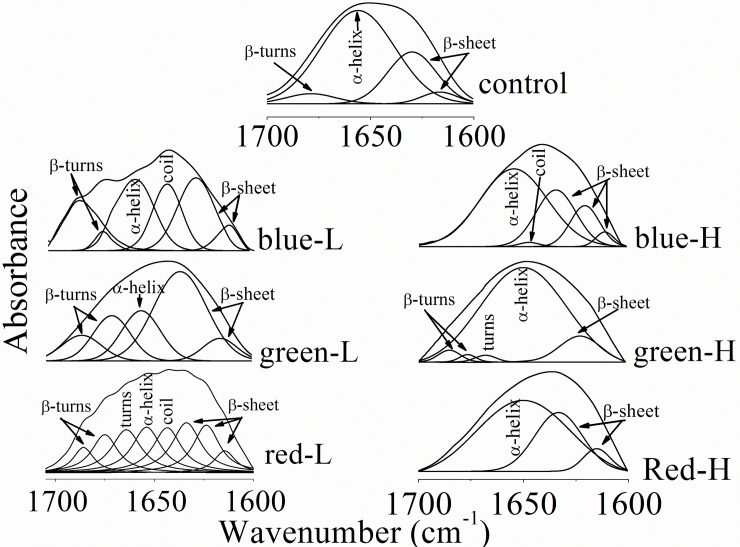
Protein secondary structure components of corneas after exposure to different color intensities.

The most sensitive spectral region to protein secondary structural components is the amide I band (1700−1600 cm^-1^), which is due almost entirely to C = O stretching vibrations of the peptide linkages [6–8]. Fig 3 shows that the contour of the control amide I band was resolved by the curve enhancement procedure into four component bands that centered around 1679 cm^-1^ (β-turns), 1656 cm^-1^ (α-helix) and two bands at 1630 cm^-1^ and 1617 cm^-1^ that correspond to β-sheet structures. Exposure to low lux of different colors was associated by increased number of the underlying structural bands in the amide I region. Regarding the low lux of the blue light; there are two β-turns structural components with a total area percentage of 27.6% (relatively higher than the normal content, 4.8%) concomitant with the presence of random coil band at 1644 cm^-1^ (17.8%). The α-helix content was markedly decreased from 66.5% in the control pattern to 24.1%. No change in the content of β-sheet was observed. Also the low-lux of the green light induce marked changes in the corneal protein amide I region, there are five structural components that indicates the presence of α-helix (19.2%), β-sheet (54.2%) and β-turns (26.6%). It is clear that the content of α-helix was reduced and both β-sheet and β-turns content were increased. The remarked changes was noticed after the exposure to the low lux of the red light where there are eight secondary structural bands were estimated and reflect the presence of β-turns (17.7%), turns (14.9%), α-helix (15.4%), random coil (14.9%) and three bands corresponds to β-sheet structure (37.1%).

The corneal protein secondary structure components were differing after the exposure to high lux of different light colors. For the blue-H, there are five structural bands and also the α-helix content was reduced (57.9%) concomitant with increased β-sheet content (41.1%) and random coil structure was detected (1%). As a result of exposing the cornea to green-H, the amide I pattern is greatly affected, the α-helical structure is dominant (84%) and β-sheet content was reduced (11%), there is no change in the content of β-turns structure, but the turns structure was detected with an area percentage of 1.3%. High lux of the red color resulted in three structural bands in the amide I region that only represent α-helix (68.9%) and β-sheet (31.3%) structures as given in Table 3.

**Table 3:** The area percentage of protein secondary structure components due to exposure to different wavelength/lux.

	Control	Blue		Green		Red	
		Low	High	Low	High	Low	High
α-helix	66.5±3.2	^†^24.1±1.6	^†^57.9±2.6	^†^19.2±2.1	^†^84±4.4	^†^15.4±3.0	68.8±1.8
β-sheet	28.7±4.1	30.5±3.7	^†^41.1±2.8	^†^54.2±4.7	^†^11±3.9	^†^37.1±1.7	31.1±2.4
β-turns	4.8±1.3	^†^27.6±1.8		^†^26.6±4.1	3.7±1.6	^†^17.7±1.0	
Turns					1.3±0.4	14.9±0.6	
Coil		17.8±2.5	1±0.04			14.9±0.8	

^†^Statistically significant.

In each cell, the first line represents the frequency and the second line reflects the bandwidth.

The detected changes in protein secondary structure so far are not only due to the variation in the individual proportions but also due to hydrogen bonding. As shown in Fig 3, the extended β-sheets (C = O out of phase, 1630 cm^-1^) and the antiparallel β-sheets (1610–1616 cm^-1^) are detected in all exposed groups to low and high lux. The changes in the protein secondary structure of the red-L group were associated with the formation of extremely strong intramolecular hydrogen bonding and/or unusual β-sheet structure (underlying peak at 1625 cm^-1^) in addition to parallel β-sheet. This strong hydrogen bonding is evident also in the blue-H exposed group. On contrary, green-H exposed group showed another different variation that is no extended-chain β-sheets.

### Fingerprint bending region

**Fig 4:**
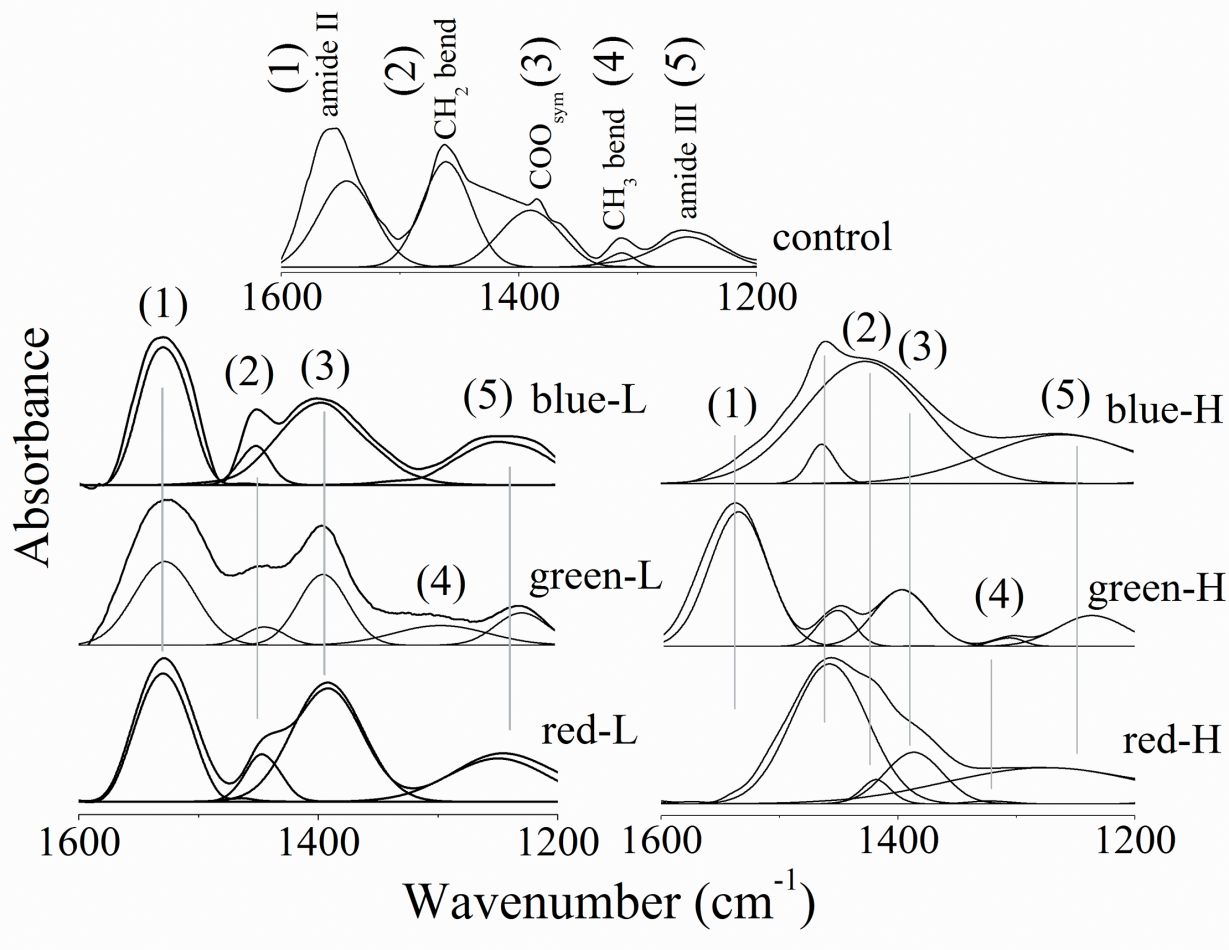
Fingerprint vibrations of corneas from all studied groups showing the underlying peaks.

In Fig 4, the fingerprint region (1600–1200 cm^-1^) and for amide II band (bending NH); the detected bands in low lux as well as green-H were characterized by reduced vibrational frequency without any change in its vibrational motion. This vibrational mode was undetected due to exposure to blue-H or red-H. On the other hand, the vibrational frequency of the CH_2_ bending band was greatly affected by the exposure to all low or high lux of different light colors involved in this study. In addition to this, cornea-CH_2_ bending band was split into two component bands that were centered at 1458 ± 1 cm^-1^ and 1418 ± 3 cm^-1^ due to exposure to blue-H and red-H. In the same context, the bandwidth of the CH_2_ bending band was markedly changed.

No change in the band position of the _sym_COO band following the exposure to low or high lux of different light colors. The main observation concerning this band is its disappearance that following the exposure to blue-H. The bandwidth showed many specific characteristics due to the exposure to low lux; increased for blue-L, decreased for green-L and unchanged for red-L (Table 4). The CH_3_ bending band was noticed only after the exposure to green light (low or high lux) with reduced band position and after the exposure to red-H (with increased band position and bandwidth) as shown in Table 4.

**Table 4:** Fingerprint region showing the bending modes of different bands and their characteristic vibrational frequency (cm^-1^) and bandwidth (cm^-1^).

	Control	Blue			Green		Red		
		Low	High		Low	High	Low	High	
Amide II	1545±1	^†^1529±1			^†^1527±3	^†^1536±1	^†^1530±2		
	44±5	44±3			54±6	46±9	49±8		
_bend_ CH_2_	1461±2	^†^1453±2	1463±3	1427±3	^†^1443±2	^†^1453±1	^†^1446±2	1458±1	1418±3
	38±2	^†^20±5	^†^17±3	102±5	^†^28±3	^†^23±4	^†^24±3	^†^62±2	19±2
_sym_COO	1390±3	1395±2			1394±3	1396±3	1392±3	1387±2	
	53±2	^†^75±8			^†^41±4	^†^45±1	56±2	^†^46±2	
_bend_ CH_3_	1315±1				^†^1296±2	^†^1308±2		^†^1323±2	
	16±3				^†^78±8	18±4		^†^31±6	
_asym_PO_2_	1258±2	^†^1244±1	1260±2		^†^1228±2	^†^1236±1	^†^1250±2	^†^1275±3	
	69±3	^†^85±2	^†^128±6		^†^41±7	^†^56±2	^†^81±4	^†^167±4	

^†^Statistically significant.

In each cell, the first line represents the frequency and the second line reflects the bandwidth.

Finally, _asym_PO_2_ band vibrational frequency was also changed as a consequence of exposure to different light intensity/color. The band position was decreased after the exposure to low lux and again; after exposure to high lux, the band position showed characteristics that depends on the light source wavelength: unchanged with blue-H, decreased for green-H and increased for red-H. Meanwhile, the bandwidth shows two contradictory behavior; it increased after exposure to blue light (low or high lux) and red light (low or high lux) while reduced for green light (low or high lux).

## Discussion

Eye is usually exposed to natural electromagnetic spectrum and man-made visual display devices. Although it has several adapted mechanisms to be protected from light exposure, certain exposure can result in temporal or permanent damage as macular degeneration [4]. Exposing eye to relatively long time-intermittent light has an impact on the cornea. Cornea -as other biological organs- is a membrane bilayer system, and the characteristics of corneal tissue is determined by the properties of this bilayer as the composition of the biopolymers and their interaction to each other as well as the way of packing together. Therefore, variation in any of these characteristics, physiologically will affect the cornea.

Amide A and Amide B bands (_asym_NH and _sym_NH of protein) are greatly affected due to different wavelengths/lux exposure, while the OH bonds vibrational characteristics of the corneal structure constituents as cholesterol indicated that changes in the microenvironment were takes place. The careful analysis of the decreased vibrational frequency of _asym_OH and _sym_OH that were shown in Fig 1 and given in Table 1, indicates that more ordered membrane structure and more vibrational motion are associated with all lux exposure except blue-L.

Deconvolution revealed that the amide I band composed of several underlying bands; the band features in these deconvoluted spectra were different. This indicates that there are changes in the type and relative proportion of the overall protein secondary structures. Low lux exposure from the blue, green or red colors induce marked changes in the corneal protein secondary structure. These light colors were associated with reduced α-helix and increased β-turns contents with the detection of other protein secondary structural components. Protein insolubility depends on the content of the β-sheet structure; more β-sheet structure resulted in more insoluble protein [9–10]. The decreased α-helix and increased β-sheet content might indicate either a structural rearrangement of the already existing proteins or expression of new types of proteins that having different structural compositions, and this is clearly observed in corneas after exposure to all lux but blue-L. Our results also indicate that after exposing corneas to blue-H or red-L lux, their protein constituents indicate an increase in both random coil and β-sheets which may give the impetus that protein are denaturated. Accordingly, low lux resulted in more folded (blue-L), insoluble (green-L) and denaturated (red-L) protein while, high lux exposure was associated with more complicated effect represented by the tendency of corneal protein to form aggregate. The red-H exposure led to losing necessary component of the protein secondary structure. The changes in the protein secondary structure were also observed in the fingerprint region (Amide II) as the bending vibrational mode of NH bond was shifted to lower frequency indicates increased random coil formation.

Analysis of the fingerprint region revealed that each color and lux has its own effect on the corneal structure; the variation in the vibrational motion of_sym_COO vibrational mode—due to amino acid side chain and fatty acids- and _asym_PO_2_ one—due to phospholipids and nucleic acids- confirms this last finding where the vibrational motion of the former one was increased for blue-L, decreased for green-L and unchanged for red-L while, for the last one it was unchanged for blue-H, decreased for green-H and increased for red-H. The lower shift in the vibrational frequency of _asym_PO_2_ indicates an increase in hydrogen bonding of phospholipids and nucleic acid.

Exposure to different blue, green or red lux was affecting the molecular packing of the acyl chain. The acyl chain packing of the lipid bilayer—i.e. the interaction of hydrocarbon chains with each other—can be monitored by the vibrational frequency of CH_2_ bending mode at 1461 cm^-1^. The splitting of this band after exposing the corneas to blue-H and red-H is indicative of an acyl chain orthorhombic or monoclinic crystal system rather than hexagonal one. The change in corneal membrane packing is also evident by the changes noticed in the CH_3_bending mode.

Molecules can rotate around single bond and in this case are defined as rotamers where, the relative amount of gauche rotamers is indicative of the level of disorder of the bilayer, and it can be calculated using the vibrational frequency of the _asym_CH_2_ mode [11–12]. The calculated gauche rotamers percentage was done according to [13], and the interesting finding is that the membrane bilayer of corneas exposed to different color/lux were characterized by higher degree of order; the population of hydrocarbon chain gauche rotamers percentage range is 0.1–0.3 compared to 0.6 in the control corneas.

## Conclusion

The detected microenvironmental changes resulted from exposing the cornea to different colors and/or lux; increased hydrogen bonding, variation in protein secondary structure component as well as variation in the acyl chain packing, were associated with higher degree of membrane bilayer order. These detected changes should have an impact on the corneal structure and its function with a potential possibility of altering its contour; hence vision impairment may results in. This last probability required further studies to determine which color and intensity may cause the most and the least pronounced changes in the corneal structure.
