# Correction: Paradox response of cornea to different color intensities of visible light: An experimental study

**DOI:** 10.1371/journal.pone.0212392

**Published:** 2019-02-11

**Authors:** Sherif S. Mahmoud, Ibrahim H. Ibrahim, Abdel Sattar M. Sallam, Ghareib W. Ali

The fourth author’s name is incorrect. The correct name is: Ghareib W. Ali. The correct citation is: Mahmoud SS, Ibrahim IH, Sallam ASM, Ali GW (2018) Paradox response of cornea to different color intensities of visible light: An experimental study. PLoS ONE 13(5): e0196827. https://doi.org/10.1371/journal.pone.0196827
